# Fibronectin Functionalization: A Way to Enhance Dynamic Cell Culture on Alginate/Hydroxyapatite Scaffolds

**DOI:** 10.3390/jfb15080222

**Published:** 2024-08-10

**Authors:** Bianca Zumbo, Benedetta Guagnini, Barbara Medagli, Davide Porrelli, Gianluca Turco

**Affiliations:** 1 Department of Medicine, Surgery and Health Sciences, University of Trieste, Piazza dell’Ospitale 1, 34129 Trieste, Italy; bianca.zumbo@phd.units.it (B.Z.); benedetta.guagnini@phd.units.it (B.G.); bmedagli@units.it (B.M.); gturco@units.it (G.T.); 2 Department of Life Sciences, University of Trieste, Via Alexander Fleming 31/B, 34127 Trieste, Italy

**Keywords:** alginate, dynamic cell culturing, fibronectin, porous bone scaffold, perfusion bioreactor

## Abstract

Bone defects are a global health concern; bone tissue engineering (BTE) is the most promising alternative to reduce patient morbidity and overcome the inherent drawbacks of autograft and allograft bone. Three-dimensional scaffolds are pivotal in this field due to their potential to provide structural support and mimic the natural bone microenvironment. Following an already published protocol, a 3D porous structure consisting of alginate and hydroxyapatite was prepared after a gelation step and a freezing-drying step. Despite the frequent use of alginate in tissue regeneration, the biological inertness of this polysaccharide hampers proper cell colonization and proliferation. Therefore, the purpose of this work was to enhance the biological properties by promoting the interaction and adhesion between cells and biomaterial with the use of Fibronectin. This extracellular matrix protein was physically adsorbed on the scaffold, and its presence was evaluated with environmental scanning electron microscopy (eSEM) and the Micro-Bicinchoninic Acid (μBCA) protein assay. The MG-63 cell line was used for both static and dynamic (i.e., in bioreactor) 3D cell culturing on the scaffolds. The use of the bioreactor allowed for a better exchange of nutrients and oxygen and a better removal of cell catabolites from the inner portion of the construct, mimicking the physiological environment. The functionalized scaffolds showed an improvement in cell proliferation and colonization compared to non-functionalized ones; the effect of the addition of Fibronectin was more evident in the dynamic culturing conditions, where the cells clearly adhered on the surface of functionalized scaffolds.

## 1. Introduction

Bone tissue, as a dynamic load-bearing tissue, serves a variety of physiological functions within the body, including body shape maintenance and bone marrow and organ protection, and possesses self-healing capabilities. While minor bone injuries often heal naturally, severe conditions related to injuries or diseases, such as fractures, bone abnormalities, tumor removal, or age-related illnesses, require surgical intervention, impacting patients’ quality of life [1]. Despite the high incidence of bone injury, treatment selection remains controversial. The common clinical treatment for bone repair is bone grafting, including autografts, allografts, xenografts, and grafts of synthetic bone materials. Although these solutions have shown to be exploitable with good outcomes [2], several drawbacks limit their use, such as availability, overall efficacy, and cost-effectiveness [3].

Tissue engineering (TE) offers a solution by developing biological substitutes that can replace damaged tissue, reducing the need for extensive surgery, with the advantages of high customization, a low risk of infection, and no evident complication. Bone tissue engineering (BTE) promotes bone regeneration, maintaining and improving tissue function and favoring cell adhesion, proliferation, and differentiation. Biological substitutes, natural or synthetic, act as temporary scaffolds, providing a supportive environment for bone growth and gradually degrading as new tissue forms. The choice of biomaterials depends on their biocompatible properties, degradation rates, and desired mechanical characteristics [4].

Alginate (Alg) is widely used because of its low cost, gelling capacity, biocompatibility, biodegradability, and good scaffold-forming properties. Derived from brown algae, Alg consists of an alternating chain of β-D-mannuronic acid (M residues) and α-D-guluronic acid (G residues). The presence of bivalent cations (e.g., Ca^2+^, Sr^2+^, and Ba^2+^ at low concentrations) leads to the formation of hydrogels thanks to the affinity of the bivalent cations with the G monomers of alginate, which trigger a cooperative process, and to the formation of crosslinkers between alginate chains [5]. Alginate has a low biological activity because it is devoid of cell-adhesive motives for cell attachment; moreover, when placed in physiological-like conditions and media, the structure hydrates and its mechanical strength and stability decrease [6,7], thus hampering its use in load-bearing bone tissue engineering. 

In order to obtain alginate scaffolds whose bioactivity and mechanical stability would be more suitable for use as bone constructs, several strategies have been explored. Bioactive materials, such as hydroxyapatite, have been introduced into the Alg structures to create composite materials [8,9,10]. The introduction of hydroxyapatite (HAp) can enhance the Alg scaffold mechanical and biological performances to better simulate the natural bone structure. HAp is the inorganic component of bone tissue and it is biocompatible and osteoconductive; indeed, thanks to the dissolution of calcium and phosphorus ions in the microenvironment, it promotes osteoblast proliferation and differentiation [11,12,13]. Alginate/hydroxyapatite (Alg/HAp) scaffolds can be prepared by the internal gelation of Alg/HAp solutions followed by a freeze-drying procedure; the obtained structure possess a highly interconnected porous network, which is a favorable environment for cell colonization and proliferation [14,15,16].

Material bioactivity is closely related to the biological interaction between cells and the surrounding material. Despite the opportunities offered by Alg/HAp scaffolds (i.e., relatively low cost of the raw materials, homogenous porosity of the final network, rapid and simple production process, to name some), they exhibit a pronounced biological inertness that hampers cell adhesion. To address this drawback, the structure can be further modified to enhance its biological properties and elicit cell adhesion; one of the most explored strategies is mimicking the extracellular matrix (ECM) environment, which is based on the conjugation with proper peptides/proteins, cell-signaling factors, and growth factors [7,17]. A few examples of this approach are the decoration of alginate with RGD (arginine, glycine, aspartic acid) peptides, Fibronectin, or growth factors [18,19]. Fibronectin (FN) is an ECM protein that acts as a key player in the communication between the intra- and extracellular environment through binding the cell membrane integrin receptors to the biomaterial surface [20]. Fibronectin can be used to functionalize biomaterials, e.g., by adsorption on their surface, improving bone cellular adhesion, migration, and spreading and increasing the deposition of the ECM and the formation of new blood vessels [21,22]. The functionalization of biomaterials through surface adsorption is generally used to avoid structural changes in the scaffold. On the other hand, the surface properties (chemical composition, roughness, and hydrophobicity, to name some) of a porous material could be altered by this process, leading to an inhomogeneous protein adsorption when exposed to the biological environment [23]. Nevertheless, numerous studies have demonstrated that proper functionalization improves cell adhesion and proliferation, enhances bioactivity, modulates the cellular response, and ameliorates the overall healing process [17,24,25]. Given the FN pivotal role in the bone regenerative process, the modulation of FN–biomaterial interaction holds significant promise for enhancing tissue regenerative responses [26,27]. 

Cell culturing on three-dimensional structures in static conditions suffers from low oxygenation, low nutrient exchange, and poor waste removal, with the consequent reduction in proliferation in the innermost part of the scaffold [28]. In addition, with this method, cells are unable to fully colonize and perfuse the scaffold. Dynamic cultures are used to improve nutrient transfer and promote scaffold colonization using the flow rate, which also stimulates the differentiation process, simulating the physiological environment [29,30].

The bioreactor system improves cell growth in a three-dimensional structure with a dynamic culture medium flow, guaranteeing the correct gas exchange between the scaffolds and the medium and allowing for the correct diffusion of metabolites and catabolites; moreover, it helps the cells uniformly colonize the porous structure [31,32]. Several studies have demonstrated that for bone regeneration, the perfusion bioreactor is the most efficient system, which allows a uniform mixing of the culturing media, better environmental control, and physical stimulation [28,33,34]. The flow rate of the medium causes internal shear stress that mimics the in vivo microenvironment, enhances cell proliferation, upregulates the expression of both osteoblastic markers, and stimulates the mineralization within the bone tissue scaffold, producing higher levels of osteogenic markers if compared to static culture conditions [29,33,34,35].

The current study aims to investigate in vitro the effects of FN functionalization on the Alg/HAp scaffolds. The presence of the protein on the constructs was evaluated quantitatively and qualitatively, and its effect was studied in terms of cell proliferation using osteoblast-like cells. Cell culturing in dynamic conditions showed the contribution of the FN in improving the interaction between the cells and the biomaterial and therefore promoting scaffold colonization.

## 2. Materials and Methods

### 2.1. Materials

Sodium alginate (Alg, F_G_ = 0.67; F_GG_ = 0.59; M_W_ = 135,000), isolated from Laminaria Hyperborea, was kindly provided by FMC biopolymers (Drammen, Norway). Phosphate-buffered saline (PBS), hydroxyapatite micrometric powder (HAp), glucono-delta-lactone (GDL), the In Vitro Toxicology Assay Kit (Resazurin-based Tox8) and Tetrazolium salts test (MTT), and Fibronectin (1 mg/mL) were bought from Merck (St. Louis, MI, USA). The μBCA assay was bought from ThermoScientific (Milan, Italy), and the ProBlue safe stain was gently provided from GiottoBiotech (Florence, Italy). Dulbecco’s Modified Eagle’s Medium (DMEM), fetal bovine serum, L-glutamine, and penicillin/streptomycin were purchased from EuroClone (Milan, Italy).

### 2.2. Preparation of Alginate/Hydroxyapatite Composite Scaffolds

Alg (2% *w*/*v*)/HAp (3% *w*/*v*) scaffolds were prepared as described by Turco and colleagues [16]. Briefly, alginate was dissolved in deionized water overnight at room temperature using a volume of water equal to 70% of the final volume of the hydrogel. Then, HAp was finely dispersed in deionized water, the suspension was stirred for 30 min, and a volume of water equal to 20% of the final volume of the hydrogel was used. The dispersed HAp was poured into the alginate solution and stirred for 30 min to achieve a homogeneous mixture. For the gelation of the solution, a solution of GDL (60 mM in a volume of deionized water equal to 10% of the final volume of the hydrogel) was added to the Alg/HAp mixture and vigorously stirred for 60 s. The mix was then poured into a 24-well plate, filling the wells. The plate was then left overnight for alginate gelation. In order to obtain the porous scaffolds, after sealing the 24-well plates with parafilm and in plastic bags, the hydrogels were frozen with a controlled freezing ramp, starting from 20 °C and decreasing to −20 °C by 1 °C every 4 min using a cryostat (28 L circulating bath, VWR, Radnor, PA, USA). The frozen hydrogels were lyophilized using an ALPHA 1-2 LD plus freeze-drier (CHRIST, Osterode am Harz, Germany) for 48 h. The dried scaffold was composed of 40% *w*/*w* alginate and 60% *w*/*w* HAp. To expose the porosity of the dried scaffolds, they were cut into cylinders (5 mm in diameter, 10 mm in height); sterilized under UV light for 90 min, changing orientation every 30 min; and washed twice for 10 min in deionized water to remove possible residues of GDL. 

### 2.3. Fibronectin Coating

A 10 µg/mL Fibronectin (FN) working solution was prepared and the scaffolds were soaked at room temperature in 2 mL of FN solution (per scaffold) for 24 h. Non-coated scaffolds, used as the control, were kept in deionized water. The scaffolds obtained are named as follows: alginate/hydroxyapatite scaffolds (Ctrl-sc) and alginate/hydroxyapatite-Fibronectin scaffolds (FN-sc).

### 2.4. Evaluation of Fibronectin Presence

#### 2.4.1. Micro-Bicinchoninic Acid (μBCA) Protein Assay

A μBCA assay (ThermoScientific, Milan, Italy) was used to detect the protein adsorbed on the scaffolds, and the protocol was modified for our purpose. Briefly, the scaffolds coated with Fibronectin were quickly washed in water to remove non-adsorbed protein and soaked in μBCA solution diluted 1:2 with water in a 96 multi-well plate. The scaffolds were incubated at room temperature for 2 h; then, the solutions were harvested and centrifuged to remove any scaffold residue, 150 μL of the supernatant was transferred in transparent 96-well plates, and the absorbance (OD = 560 nm) was measured. A scaffold without Fibronectin was used as the control. The Fibronectin presence was also tested after 24 h of incubation of the scaffolds in PBS in order to verify the coating stability.

#### 2.4.2. Attenuated Total Reflectance–Fourier Transform Infrared (ATR-FTIR) Spectroscopy

Ctrl-sc and FN-sc were tested with infrared spectroscopy in order to analyze the presence of Fibronectin and HAp within the structures. The infrared spectra were collected using a Nicolet iS50 spectrometer (ThermoScientific, Milan, Italy) by measuring the transmittance in the wavenumber range of 4000–525 cm^−1^. Three samples for each condition were analyzed, collecting 32 scans for each spectrum with a resolution of 0.482 cm^−1^.

### 2.5. Biological Tests on Scaffolds

Human MG-63 osteoblast-like cells (ATCC number: CRL-1427) were used to test the biological properties of the scaffolds. The cells were cultured on DMEM High-Glucose medium (Dulbecco’s Modified Eagle’s Medium), supplemented with 2 mM l-glutamine, 10% *v*/*v* fetal bovine serum (FBS), 100 U penicillin, 0.1 mg/mL of streptomycin, into a 25 cm^2^ culture flask at 37 °C in a humified 5% CO_2_ atmosphere. The scaffolds were left for 24 h in DMEM High-Glucose medium to equilibrate. Scaffolds were seeded with a suspension of 4 × 10^5^ cells in 40 µL of cell medium and incubated at 37 °C in a humidified 5% pCO_2_ atmosphere. After 4 h of incubation, 2 mL of culture medium was slowly poured in each well, and the scaffolds were incubated at 37 °C in a humidified 5% pCO_2_ atmosphere, replacing the medium every 3 days. After 24 h of incubation, Alamar Blue was used to evaluate cell adhesion, while the proliferation was tested for 10 days using Alamar Blue and the MTT assay. 

#### 2.5.1. Dynamic Culture in Bioreactor

**Figure 1:**
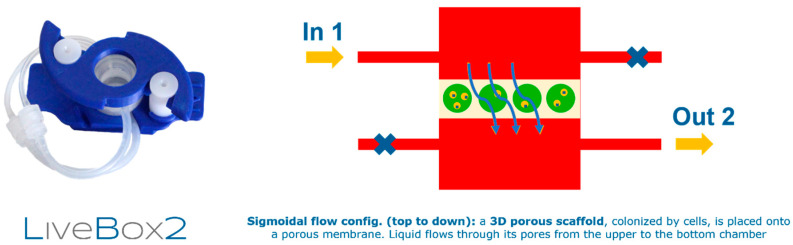
Schematic representation of the Live Box 2 culture system used in this study. Reproduced with permission of IVTech.

MG-63 cells were cultured on FN-sc scaffolds in a perfusion bioreactor (IVTech, Pisa, Italy) to evaluate cell viability under the effects of a dynamic culture system. MG-63 cells were seeded on top of the scaffold (4 mm in height, 5 mm in diameter, 2 × 10^5^ cell/scaffolds in 40 μL of DMEM High-Glucose medium), maintained on a 24 well-plate, and incubated for 4 h at 37 °C and 5% CO_2_ in static conditions to allow adhesion to the structure, after which 2 mL of medium was added. A period of 24 h after seeding, samples were placed in the bioreactor chambers of LiveBox 2 (Figure 1) and connected to the bioreactor circuit, and the flow rate was set to 0.15 mL/min for three days and then to 0.2 mL/min for the remaining course of the experiment. During this period, the bioreactor was located inside of a biological incubator at 37 °C and 5% CO_2_.

#### 2.5.2. Alamar Blue Assay

The Alamar Blue assay was used to quantitatively measure the metabolic activity of living cells on the scaffolds. At the specific time points, the culture medium was removed and the samples were moved into a new 24-well culture plate to remove cells adhered to the old culture plate. An amount of 700 µL of a solution of Alamar Blue (Merck, St. Louis, MI, USA) diluted 1:30 in DMEM High-Glucose medium was then added to each scaffold, which were then incubated for 4 h at 37 °C and in 5% CO_2_ in dark conditions. After incubation, 200 µL of culture medium, for each sample, was transferred to a 96-well black plate, and the fluorescence intensity (excitation wavelength: 544 nm; emission wavelength: 590 nm) was measured using a GloMax Multi+ Detection System (Promega Corporation, Madison, WI, USA). The fluorescence of the Alamar solution incubated with an empty scaffold was used as the background. After the measurements, the scaffolds were gently washed with phosphate-buffered saline (PBS) to remove the Alamar Blue solution and 2 mL of fully supplemented DMEM was added to each well. The proliferation rate for each sample was calculated as the ratio between the fluorescence read at each time point and the fluorescence of the same scaffold read on day 1. 

#### 2.5.3. MTT Assay

The cell metabolic activity and the cell colonization of the scaffolds were evaluated using the MTT test (Tetrazolium salts test, Merck, St. Louis, MI, USA). An amount of 700 µL of a solution of MTT in DMEM High-Glucose medium (1 mg/mL) was added to each scaffold after the medium was removed, and the scaffolds were then incubated for 4 h at 37 °C in 5% CO_2_ in dark conditions. To evaluate the cell colonization, images of the formazan crystal cell clusters were detected by a stereoscope and subsequently with a scanning electron microscope working in environmental conditions (eSEM). For the cell proliferation analysis, the purple formazan crystals were dissolved in 2 mL of dimethyl-sulfoxide (DMSO), shaking the samples at room temperature for 30 min. To quantitatively evaluate the amount of formazan, the supernatants were transferred to Eppendorf tubes and centrifuged to eliminate scaffold residues. An amount of 200 μL of each supernatant was transferred to a 96-well transparent plate and the absorbance (OD = 560 nm) was measured. The absorbance reads from scaffolds without cells were used as blanks. 

### 2.6. Cell Morphology Study by ESEM Observation

Scaffolds were examined by scanning electron microscopy (Quanta250 SEM, FEI, Hillsboro, OR, USA). After air-drying for 30 min, a razor blade was used to expose various planes of the Ctrl-sc and FN-sc in order to analyze the inner structure in addition to their surface. The scaffolds were placed on aluminum stubs which were previously coated with carbon double-sided tape to help the conductivity. The scaffolds were analyzed in environmental conditions with SEM to analyze their structure and morphology. The working distance was tuned to obtain a suitable magnification and the acceleration voltage was set to 30 kV.

### 2.7. Stereoscope Imaging

After 4 h of incubation, the scaffolds were sectioned at various planes and viewed by a stereomicroscope (Leica MZ16, Leica, Wetzlar, Germany) at different magnifications. Image processing and analysis were performed by Image Pro Plus software (version 6.2, Media Cybernetics, Rockville, MD, USA) and Fiji software (version 2.13.0) was used to set a scale bar. 

### 2.8. Statistical Analysis 

Statistical analyses were performed using GraphPad software (version 8.0.2, Insight Partners, New York, NY, USA). The Shapiro−Wilk test was used to test the normality of the data; the ANOVA test with Bonferroni’s correction was used to test data which satisfied the normality assumptions; and the Kruskal−Wallis and Mann−Whitney non-parametric tests, applying Bonferroni’s correction, were used to test data which did not satisfy the normality assumptions. Statistical significance was set at α = 0.05.

## 3. Results

The material was obtained by freeze-drying alginate/hydroxyapatite hydrogels, prepared using an internal gelation method [16]. The method employed for the gelation exploits the partial ionization of HAp due to the acidification of the hydrogel induced by the slow hydrolysis of the GDL, and the amount of GDL added to the Alg/HAp mixture guarantees that only a small fraction of HAp is ionized and frees the Ca^2+^ ions which are needed for the gelation of alginate [15,16]. X-Ray Diffraction analyses have already proved that after the formation process of the scaffolds, the HAp contained in the structures maintains its chemical properties and integrity [16]. The material was functionalized by Fibronectin adsorption to increase biological properties. The optimization of Fibronectin concentration was evaluated qualitatively with an MTT assay (Figure S1).

### 3.1. Fibronectin Coating Characterization

**Figure 2:**
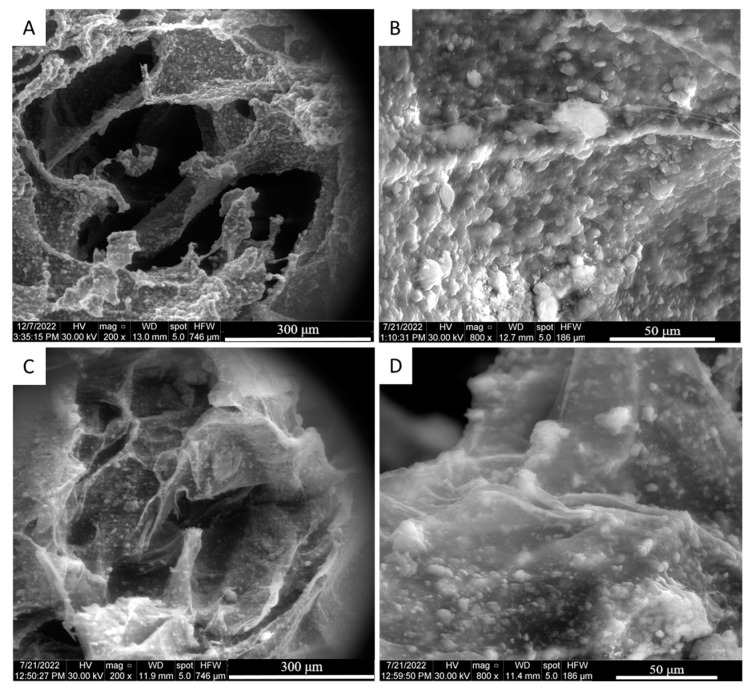
Structural characterization of scaffold. eSEM analysis of Ctrl-sc (**A**,**B**) and FN-sc (**C**,**D**). There are different magnifications in each panel; from left to right: 200×; 800×.

The adsorption of Fibronectin on the scaffold structure was studied qualitatively after 24 h of adsorption through eSEM analysis. The imaging was performed on the cross-section of both the Ctrl-sc and FN-sc scaffold (Figure 2). The images for Ctrl-sc (A and B) and FN-sc (C and D) show the rough surface of the scaffold trabeculae, confirming the presence of hydroxyapatite in both preparations. The presence of Fibronectin can be appreciated as a thin translucent layer covering the surface of the scaffold, as it is shown in Figure 2C,D.

**Figure 3:**
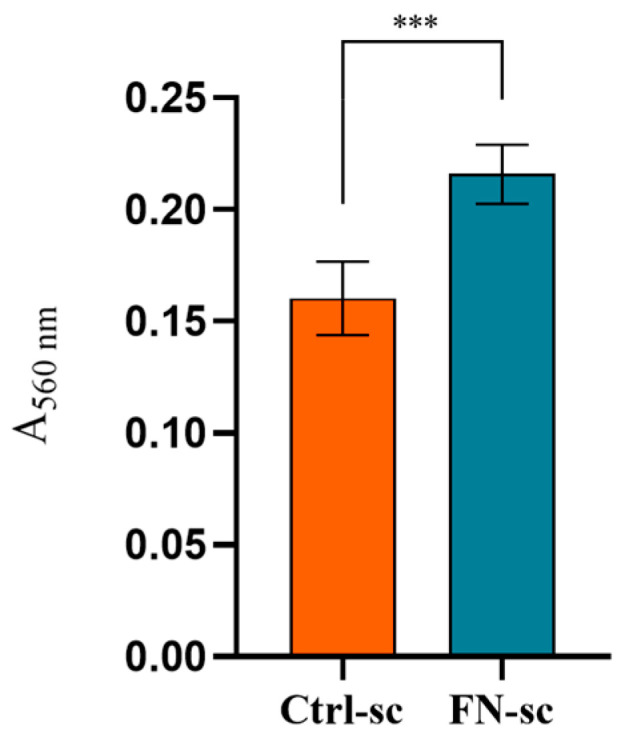
μ-BCA results of Ctrl-sc and FN-sc read in absorbance at 560 nm. Error bars represent the standard deviation calculated on the mean of 6 scaffolds for each condition. Statistical analysis was performed using the Mann–Whitney test for comparison between the two groups, applying Bonferroni’s correction. Statistically significant differences are indicated as asterisks (*). *** = *p* < 0.001. N = 6.

Further analyses to confirm the presence of Fibronectin, after functionalization, were conducted using a μ-BCA protein assay. The results shown in Figure 3 demonstrate a significant difference in the absorbance between Ctrl-sc and FN-sc, confirming the presence of Fibronectin, as well as the stability of the coating after 24 h of incubation in PBS, later confirmed by the μ-BCA protein assay and Coomassie blue staining, as reported in Supplementary Materials (Figures S2 ad S3). As reported in Figure S4 of the Supplementary Materials, the FT-IR analysis was unable to detect the presence of Fibronectin due to its low concentration. 

### 3.2. Biocompatibility and Cell Morphology on Scaffolds

**Figure 4:**
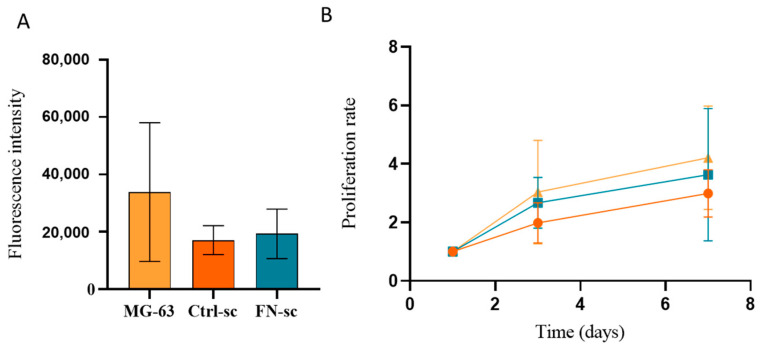
Biocompatibility of Ctrl-sc (●) and FN-sc (■) evaluated in terms of MG-63 adhesion and proliferation and compared with MG-63 (▲) cultured in wells. Fluorescence intensity of Alamar Blue assay measured to evaluate cell adhesion (**A**) and cell proliferation within the scaffold (**B**). Error bars represent the standard deviation calculated on the mean of 3 scaffolds at each time point.

MG-63 cell proliferation in the Fibronectin-functionalized scaffolds was evaluated to investigate the biological effects of the Fibronectin coating. The evaluation of cell adhesion by the Alamar Blue test (Figure 4A) did not show any statistically significant difference when the cells were cultured in the Ctrl-sc or in the FN-sc. The proliferation rates, calculated by normalizing the fluorescence reads at each time point with respect to day 1, were also compared with control cells grown on the surface of a multi-well plate. Cell proliferation data, reported in Figure 4B, show that both Ctrl-sc and FN-sc are able to sustain cell colonization and proliferation with a slight improvement in FN-sc if compared with Ctrl-sc, although the differences are not statistically significant.

**Figure 5:**
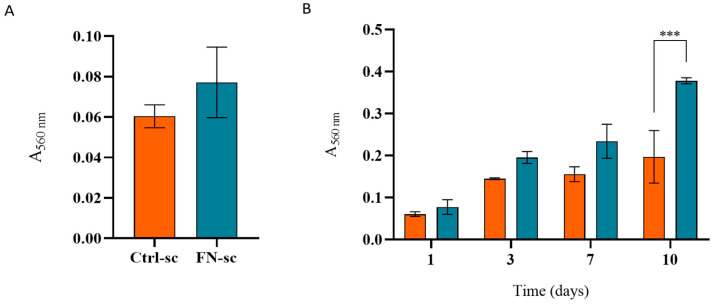
Cell adhesion (**A**) and viability (**B**) on Ctrl-sc (orange) and FN-sc (light blue) evaluated with the MTT assay; error bars represent the standard deviation calculated on the mean of 3 scaffolds at each time point. The statistical analysis was performed with the ANOVA test, applying Bonferroni’s correction. Statistically significant differences are indicated with asterisks (*). *** = *p* < 0.001. N = 3.

Furthermore, the MTT test (Figure 5) was used to assess the cell viability. In comparison to the Alamar analysis, on day 1 (A), the absorbance values are similar between the two conditions, whereas over time (B), the MTT test shows a statistically significant increase in cells grown in FN-sc compared to Ctrl-sc, after 10 days of culture.

**Figure 6:**
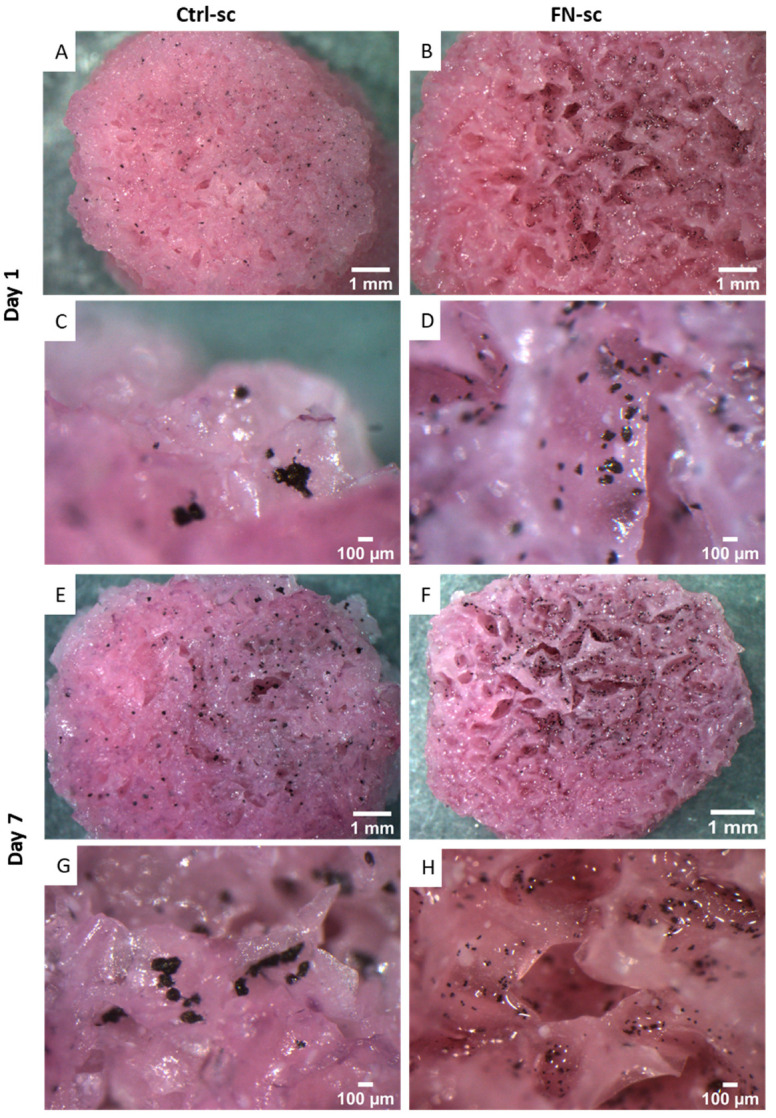
Top view of Ctrl-sc and FN-sc of MG-63 on day 1 (**A**–**D**) and 1 week (**E**–**H**) after seeding, at two different magnifications.

The MTT assay was also used as a qualitative assay; in fact, the insoluble formazan crystals allowed the identification of purple clusters corresponding to viable cell clusters. In particular, on day 1 and day 7 after seeding, the results of the viability test were compared with the images obtained using a stereoscope (Figure 6) on both scaffolds. The images show that on the functionalized scaffold, the MG-63 cell population forms smaller cell clusters uniformly distributed all over the surface, compared to the Ctrl-sc, where the cell clusters are less distributed, mainly on the external surface. At a higher magnification (C and D), cell clusters on day 1 are notably larger and less numerous on Ctrl-sc, whereas in functionalized scaffolds, they are smaller and colonize the surface better, thus indicating that the cells are better distributed on Fibronectin-functionalized scaffolds.

On the 10th day of cell culture, a viability test was performed and the stereoscope analysis confirmed what was previously seen in adhesion. There was better colonization of the scaffold in the presence of Fibronectin, and MG63 clusters were uniformly distributed over the surface and were smaller compared to the cells in the control scaffolds, where the cells created isolated clusters (G and H), probably due to the inertial nature of the scaffold.

**Figure 7:**
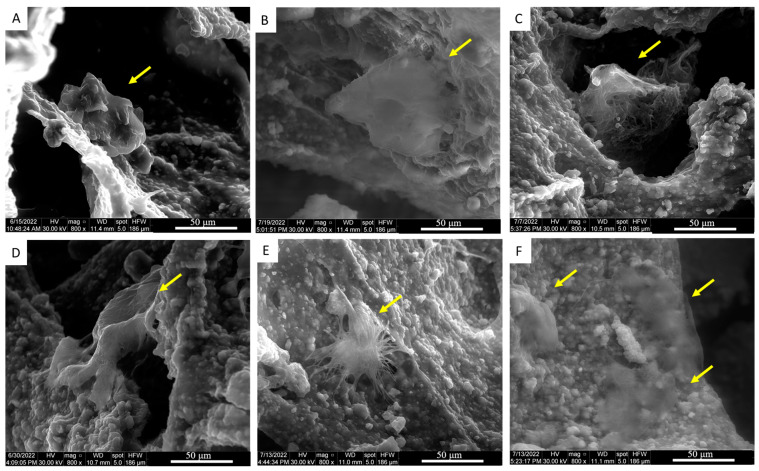
eSEM micrograph of cell (indicated by yellow arrows) seeded on Ctrl-sc (**A**–**C**), with a round shape morphology, and on FN-sc (**D**–**F**), with a well-spread morphology.

The cell morphology on the scaffold surface was studied by environmental SEM analysis (Figure 7); after one week of culture, the MG-63 cells in adhesion on Ctrl-sc and on FN-sc can be distinguished thanks to their smooth surface compared to the rough scaffold surface. Cells on Ctrl-sc (A; B; C) did not appear to be well spread, as already seen, and exhibited a round shape morphology, while those on the FN-sc ones (D; E; F) appeared to adhere more favorably, displaying a spread morphology.

The presented data consistently support the hypothesis that functionalization with Fibronectin has an interaction effect between cells and the scaffold. These results were obtained in static culture conditions on the three-dimensional porous structure maintained on 24-well plates, which is the most common method used to realize cell–scaffold constructs. However, the static culture possesses some limitations related to the absence of a nutrient transport and waste elimination system, which often causes high apoptosis in the core of the structure [28]. Dynamic cultures using bioreactors are used to alleviate this nutrient transfer limitation by continuously mixing media and by convectively transporting nutrients to cells seeded on 3D scaffolds. The mechanical stress created in the bioreactor microenvironment also helps in the differentiation of bone cells simulating in vivo conditions.

**Figure 8:**
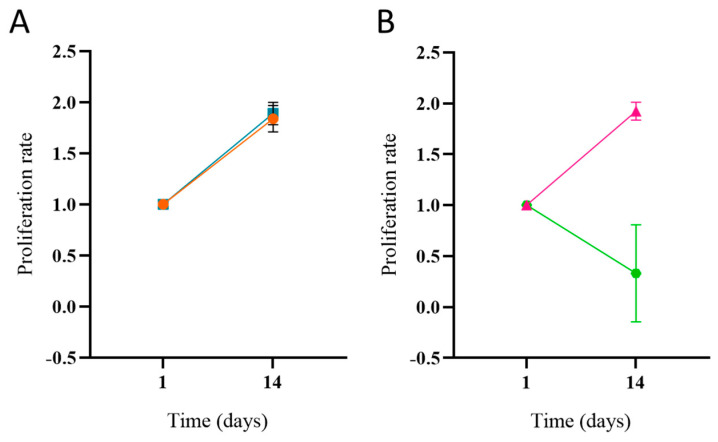
Proliferation of MG-63 on Ctrl-sc in static ((**A**), ●) and dynamic ((**B**), ●) conditions, and FN-sc in static ((**A**), ■) and dynamic ((**B**), ▲) conditions, using a perfusion bioreactor. Error bars represent the standard deviation on the mean of 3 scaffolds.

Based on the assumption that Fibronectin facilitates cell adhesion, cell viability was evaluated in dynamic conditions, hypothesizing that the protein is capable of allowing a reduction in cell detachment due to the flow rate of the bioreactor system. In this condition, the cell culture medium flow was activated after 24 h from cell seeding to allow stable cellular adhesion to the substrate. Cell adhesion was evaluated the day after activation of the dynamic medium flow and the proliferation was then evaluated after 14 days from seeding, with the Alamar Blue assay. The proliferation graph (Figure 8) illustrates a comparable proliferation rate between static and dynamic conditions in the presence of the Fibronectin, while on the control scaffolds, cells underwent a decrease in the proliferation rate over time when cultured in dynamic conditions.

**Figure 9:**
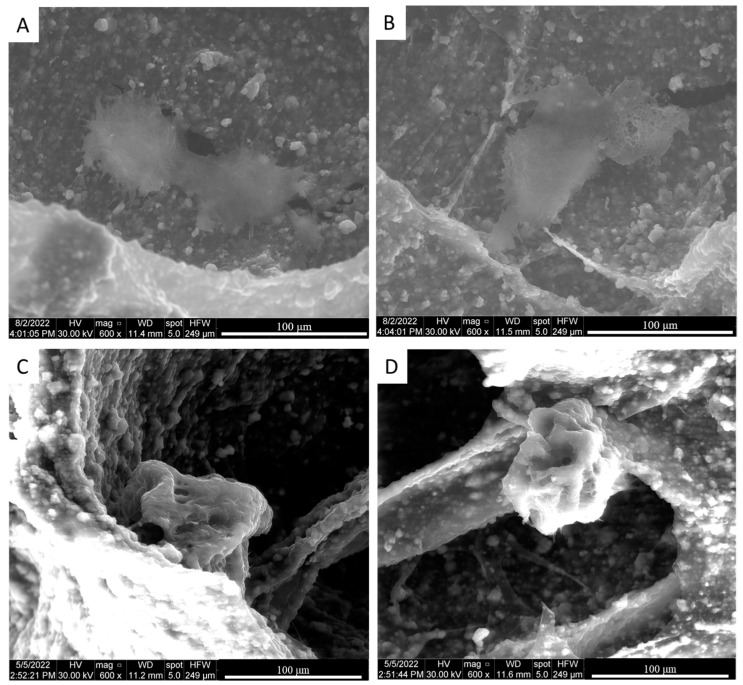
eSEM micrographs of cells spread on FN-sc (**A**,**B**) and Ctrl-sc (**C**,**D**) after 14 days in a dynamic culture with a perfusion bioreactor.

To support the data, micrographs acquired with eSEM (Figure 9) show the cells well adhered and spread on the Fibronectin-functionalized scaffold surface after 14 days of dynamic culture (A; B), while the morphology of the cells cultured in Ctrl-sc appears more round-shaped, proving that the cells were not well adhered and spread if compared to the ones cultured on FN-sc (C; D). 

## 4. Discussion

In bone regeneration, the optimal tissue repair requires a three-dimensional biocompatible scaffold which can ensure cell adhesion, colonization, and proliferation [36]. In this work, three-dimensional porous scaffolds were prepared with alginate and hydroxyapatite and subsequently functionalized with Fibronectin. The purpose was to evaluate the effects of Fibronectin on a well-characterized structure [15,16], focusing on the bioactivity of the system, as well as the possible application in a dynamic system, with a perfusion bioreactor, to mimic the physiological environment. To avoid structural modification due to the covalent binding, Fibronectin was physically adsorbed onto the scaffold trabeculae [22]. The μ-BCA analysis (Figure 3) and eSEM micrograph (Figure 2) confirmed not only the successful protein adsorption but also the unaltered structure of the scaffold overall. 

The biological properties of FN-sc were assessed using MG-63 osteoblast cells, which are commonly used for in vitro analysis of bone implants [37]. The adhesion and proliferation were evaluated with two assays commonly used for cell culture and in tissue engineering studies: Alamar Blue and MTT assays. Alamar Blue, although non-toxic, has limitations in detecting cells in 3D structures, with an underestimation of cell viability [38]; despite the fact that it can be useful for monitoring cell viability over time. The MTT assay is non-viable but provides a more accurate correlation between cells and absorbance values obtained; furthermore, the insoluble formazan crystals allowed the imaging of the cell clusters to evaluate the cell distribution in the scaffold. The development of a quantitative assay for viable cell estimation for a 3D scaffold was one of the crucial points of this work.

In the adhesion, there were no significant differences between the samples, but over time, a higher proliferation of the cells on FN-sc was visible. Stereomicroscope analysis (Figure 6) and eSEM micrographs of Ctrl-sc (Figure 7) show round-shaped cells and large and non-uniform cell clusters, demonstrating poor homogeneity in the cell colonization. In comparison, FN-sc reveals smaller and more dispersed clusters, which corresponds to a better distribution of cells all over the surface. After 7 days of culture, the effects of Fibronectin become extremely relevant as the cells on FN-sc appear to grow on the trabecular surface with the morphological features of a well-adhered cell. The data underline the contribution of Fibronectin in facilitating cell distribution and proliferation.

To tackle issues like cell apoptosis in the inner parts of three-dimensional scaffolds or inefficient waste removal, scientists utilize perfusion bioreactors. These specialized systems mimic the in vivo conditions, facilitating better nutrient transfer and more effective cell colonization of the scaffold [28]. It has been demonstrated that for bone regeneration, the perfusion bioreactor is the most efficient system, which allows for a good oxygenation and transport of nutrients and metabolites, and it maintains a uniform cell distribution and survival within the graft [28,33]. 

In a perfusion system, the flow rate of the medium plays an important role because it causes internal shear stress that mimics the in vivo microenvironment, enhances cell proliferation, and upregulates the expression of osteoblastic markers, but it can also hamper cell adhesion, causing cell detachment from the 3D scaffolds. Therefore, the purpose of using Fibronectin is also to improve the colonization of the scaffold under dynamic conditions.

The flow rate of the medium plays an important role because it can produce a shear stress sufficient to detach the cells from the 3D scaffold. After seeding, the cell-loaded scaffolds were maintained in static conditions for 24 h to allow the cells to adhere well to the surface of the scaffold to prevent detachment. Subsequently, the medium flow rate was applied to the scaffolds and was gradually increased over the following days. 

The 0.2 mL/min flow rate was first chosen to understand how mechanical stimulation influences cell proliferation on the scaffolds; this flow rate should be enough to avoid possible mechanical stress, such as compression, that can derive from high pressure on the scaffold structure.

After 14 days of culture, the data demonstrate that the presence of Fibronectin is crucial for successful adhesion and proliferation of the cells on the 3D structure under a medium flow. In fact, the cells are not proliferating on Ctrl-sc in dynamic conditions, although with an initial adhesion, and are also detached from the scaffold surface by the flow. The cells on the FN-sc show a behavior similar to the same scaffold under static cell culturing conditions. We can speculate that the presence of Fibronectin aids in preventing cell detachment and, therefore, also promotes a better colonization in the innermost part of the scaffold. In the future, more extensive experiments will be performed to better explore the potential of a perfusion bioreactor system and its contribution to the cell differentiation process; investigating the molecular mechanism underlying cell–scaffold interactions will provide deeper insights into the functionalization process, potentially leading to more effective bone tissue engineering strategies. Moreover, future aims are to better investigate the interactions between the cells and the scaffolds, and how the surface properties affect the cell adhesion, colonization, and differentiation of the cells.

## 5. Conclusions

In the present work, alginate/hydroxyapatite porous scaffolds were successfully functionalized with Fibronectin to overcome the biological inertness of an alginate composite scaffold. An evaluation of the efficacy of the functionalization process and the influence of Fibronectin on cell response was carried out. This study demonstrates the positive impact of Fibronectin on MG-63 cell behavior, leading to a higher proliferation and improving scaffold colonization if compared to control samples cultured in static conditions. To reduce the drawbacks of static cultures, such as cell apoptosis in the innermost part of the scaffold, or the lack of a waste removal system, it was decided to evaluate proliferation in dynamic conditions in a perfusion bioreactor. The enhanced cell responses were particularly evident, revealing the pivotal role of Fibronectin in influencing cellular interactions with three-dimensional scaffolds. Given the results obtained, future studies will focus on the exploration of Fibronectin-functionalized alginate/hydroxyapatite scaffolds, evaluating optimal dynamic conditions to develop a system with practical solutions for addressing bone defects and analyzing the effects on cell differentiation and bone tissue formation.

## Data Availability

The raw data supporting the conclusions of this article will be made available by the authors upon request.

## Supplementary Materials

The following supporting information can be downloaded at https://www.mdpi.com/article/10.3390/jfb15080222/s1, Figure S1. Three concentrations: 5 μg/mL (A,B); 10 μg/mL (C,D); 20 μg/mL (E,F) of Fibronectin were tested to identify the suitable condition. The panel shows the effects of Fibronectin on MG-63 growth (day 7) at two different magnifications: 1× (A,C,E) and 4× (B,D,F). Figure S2. Ctrl-sc (A) and FN-sc (B–D) treated with Coomassie Blue staining. Figure S3. A: μ-BCA results of FN-sc as prepared (T0) and after 24 h of incubation in PBS (T1),read in absorbance at 560 nm. Error bars represent the standard deviation calculated on the mean of 6 scaffolds for each condition. B–E: FN-sc treated with Coomassie Blue staining, after 24 h of incubation on PBS. Figure S4. ATR-FTIR spectra of Fn-sc compared with Ctrl-sc.
