# Supplementary material for: Fibronectin Functionalization: A Way to Enhance Dynamic Cell Culture on Alginate/Hydroxyapatite Scaffolds
Source: J Funct Biomater. 2024 Aug 10;15(8):222. doi: 10.3390/jfb15080222 (PMC11355923; doi:10.3390/jfb15080222)
Supplement: Supplementary file 1 [file jfb-15-00222-s001.zip › jfb-3120778-supplementary.pdf]

Fibronectin Functionalization: A Way to Enhance Dynamic Cell Culture on Alginate/Hydroxyapatite Scaffolds

Supplementary Materials

Optimization of Fibronectin concentration

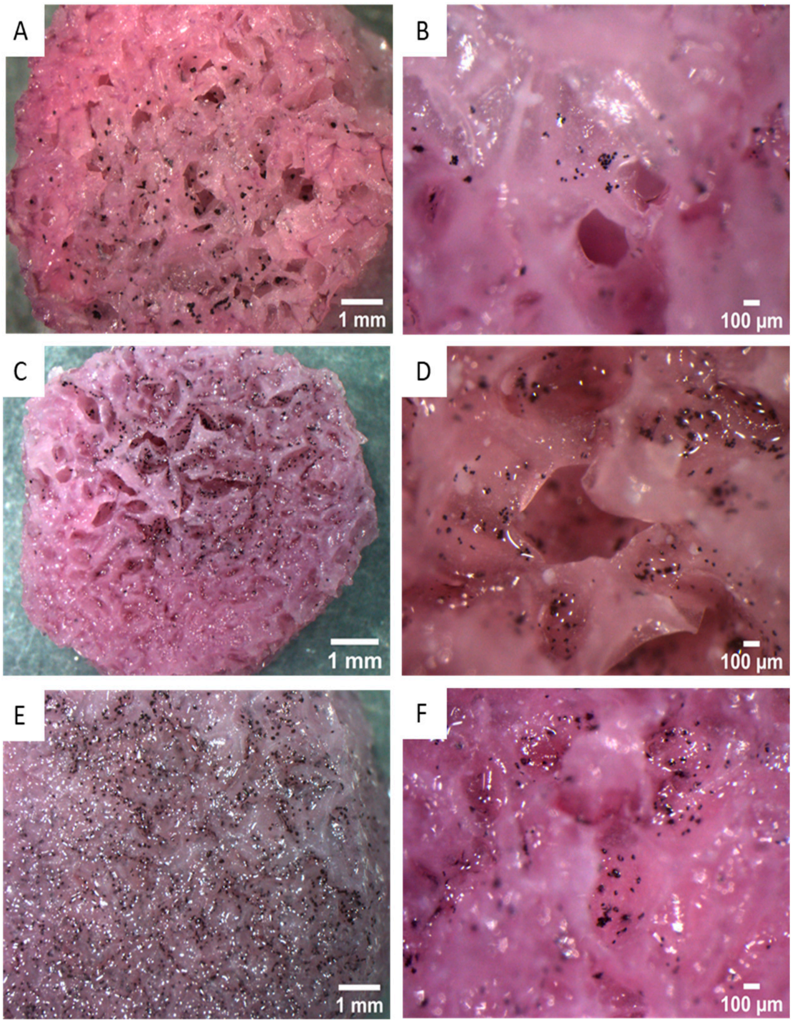

Figure S1. Three concentrations: 5 µg/mL (A; B); 10 µg/mL (C; D); 20 µg/mL (E; F) of Fibronectin were tested to identify the suitable condition. The panel shows the effects of Fibronectin on MG-63 growth (day 7) at two different magnifications: 1x (A; C; E) and 4x (B; D; F).

Fibronectin characterization

Coomassie blue staining

The ProBlue safe stain (GiottoBiotech, Italy) was used to label the Fibronectin after the functionalization. The scaffolds were gently washed in water and then soaked with 1 mL of staining for 3 minutes. Then samples were washed four times for 10 minutes with warm deionized water to eliminate exceeding dye. A last washing step in deionized water was performed overnight under agitation. Scaffolds without Fibronectin were used as controls. The next day scaffold dye was evaluated through stereoscope imaging.

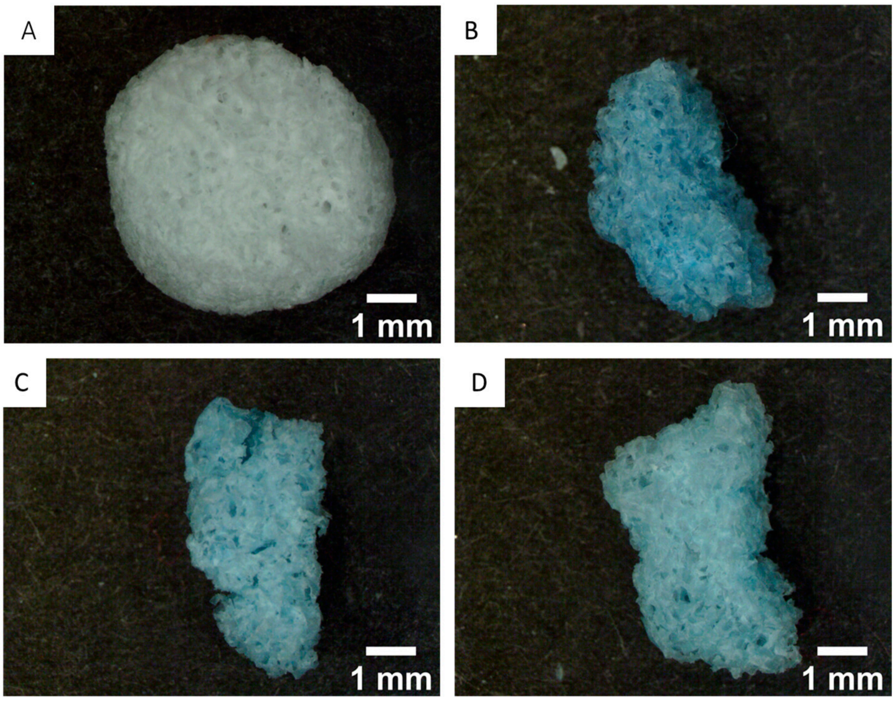

Figure S2. Ctrl-sc (A) and FN-sc (B; C; D) treated with Coomassie Blue staining.

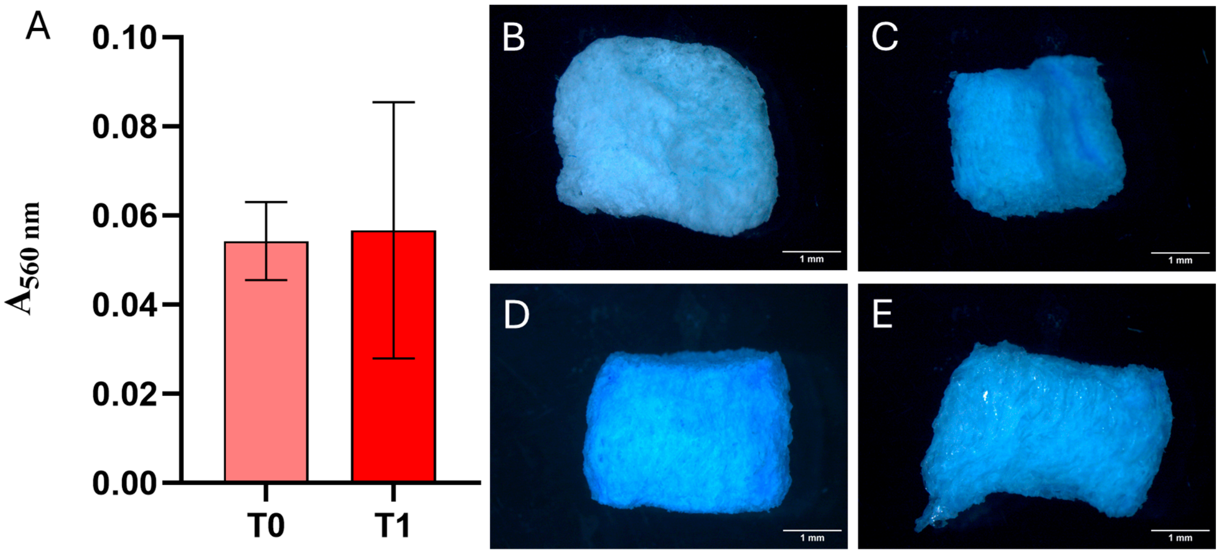

Figure S3. A: µ-BCA results of FN-sc as prepared (T0) and after 24 hours of incubation in PBS (T1),read in absorbance at 560 nm. Error bars represent the standard deviation calculated on the mean of 6 scaffolds for each condition. B-E: FN-sc treated with Coomassie Blue staining, after 24 hours of incubation on PBS.

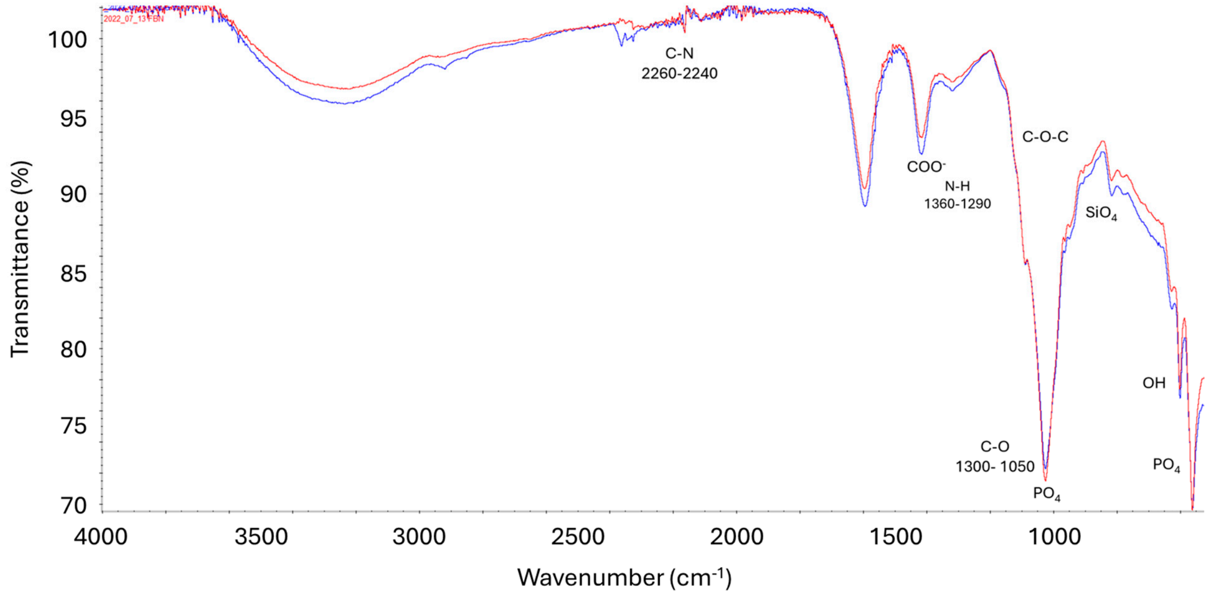

Figure S4. ATR-FTIR spectra of Fn-sc compared with Ctrl-sc.
